# Topically Delivered Adipose Derived Stem Cells Show an Activated-Fibroblast Phenotype and Enhance Granulation Tissue Formation in Skin Wounds

**DOI:** 10.1371/journal.pone.0055640

**Published:** 2013-01-31

**Authors:** Seok Jong Hong, Sheng-Xian Jia, Ping Xie, Wei Xu, Kai P. Leung, Thomas A. Mustoe, Robert D. Galiano

**Affiliations:** 1 Department of Surgery/Division of Plastic and Reconstructive Surgery, Laboratory for Wound Repair and Regenerative Medicine, Feinberg School of Medicine,Northwestern University, Chicago, Illinois, United States of America; 2 Microbiology Branch, US Army Dental and Trauma Research Detachment, Institute of Surgical Research, Fort Sam Houston, Texas, United States of America; New York Medical College, United States of America

## Abstract

Multipotent mesenchymal stem cells (MSCs) are found in various tissues and can proliferate extensively *in vitro*. MSCs have been used in preclinical animal studies and clinical trials in many fields. Adipose derived stem cells (ASCs) have several advantages compared to other MSCs for use in cell-based treatments because they are easy to isolate with relative abundance. However, quantitative approaches for wound repair using ASCs have been limited because of lack of animal models which allow for quantification. Here, we addressed the effect of topically delivered ASCs in wound repair by quantitative analysis using the rabbit ear model. We characterized rabbit ASCs, and analyzed their multipotency in comparison to bone marrow derived-MSCs (BM-MSCs) and dermal fibroblasts (DFs) *in vitro*. Topically delivered ASCs increased granulation tissue formation in wounds when compared to saline controls, whereas BM-MSCs or DFs did not. These studies suggest that ASCs and BM-MSCs are not identical, though they have similar surface markers. We found that topically delivered ASCs are engrafted and proliferate in the wounds. We showed that transplanted ASCs exhibited activated fibroblast phenotype, increased endothelial cell recruitment, and enhanced macrophage recruitment *in vivo*.

## Introduction

Wound repair is a complex and dynamic process which consists of inflammation, angiogenesis, and tissue formation and remodeling [1], [2], [3]. Upon injury, fibrin clots are deposited on the wound site to prevent hemorrhage. Circulating platelets migrate to the wound and release inflammatory signals such as transforming growth factor-β (TGF-β), platelet-derived growth factor (PDGF), and epidermal growth factor (EGF). This is followed by the infiltration of neutrophils and macrophages and the migration of keratinocytes to the wound to recover the barrier function of skin. Endothelial cells and fibroblasts migrate to the site and build up granulation tissues by depositing collagen and other extracellular matrices. During the final stages of repair, fibroblasts remodel the collagen by producing matrix metalloproteinases (MMPs) over a course of several months. Thus wound repair is a highly orchestrated sequential process in which signals of one cell type regulate other cell types in a cascade.

Growth factors have been used to improve the clinical outcome of the wound repair process. It has become clear that the use of growth factors, specifically as single-agent therapies, has limited impact on wound repair [4]. This could be partly due to the rapid degradation of therapeutic growth factors in the wound site or the requirement for the administration of these growth factors in a proper spatiotemporal sequence in order to improve wound repair. Recent progress in regenerative medicine has suggested multipotent stem cells or progenitor cells for tissue repair [5], [6], [7], [8]. It is thought that the transplanted stem cells or progenitor cells can integrate themselves to the environment and control the wound repair process by secreting factors and communicating with other cells to improve the clinical outcome of wound repair. In addition, stem cells could mediate wound repair by replacing damaged tissue by both differentiating into the required cells and inducing surrounding cells to dedifferentiate to replace the tissues.

MSCs have been isolated from various tissues such as bone marrow, adipose tissue, umbilical cord blood, skeletal muscle, and brain [7], [9]. MSCs have ability to attach to plastic to form fibroblast-like colonies and to proliferate extensively *in vitro*. MSCs can be differentiated into multiple lineage cells: chondrocytes, cardiomyocytes, adipocytes, osteoblasts, endothelial, and neuronal cells [7], [10], [11], [12], [13]. Among MSCs, ASCs can be easily obtained in large quantities with minimal morbidity and invasiveness [14], [15], [16]. ASCs activate repair processes in a paracrine manner by secreting cytokines and growth factors, such as vascular endothelial growth factor (VEGF), TGF-β, granulocyte/macrophage colony stimulating factor (GM-CSF), stromal derived factor 1 (SDF-1), and hepatocyte growth factor (HGF) [13], [17], [18], [19]. These cells also recruit endogenous stem (or progenitor) cells and can stimulate them to differentiate into the required cell types. ASCs suppress immune reactions and have reduced histocompatability antigens [20], [21], [22], [23]. ASCs have been used in preclinical animals studies and clinical trials in the field of reconstructive surgery, orthopedics, and immune diseases [7], [15], [24], [25].

Many animals - such as mouse, rat, rabbit, and pig - have been used in wound healing studies. However, there is no ideal model which exactly resembles human wounds. For example, open wounds in rodents heal quickly primarily due to wound contraction because of the subcutaneous panniculus carnosus muscle, which is not characteristic of human wounds. Rather, human skin wounds heal to a significant degree by generation of new tissue (granulation tissue and re-epithelialization) in addition to contraction. Thus, though ASCs are a promising candidate for cell therapy in wound repair, there have been limitations in the quantitative analysis of wound repair [26], [27], [28], [29]. The rabbit ear model has a unique advantage in wound healing study because of the ability to evaluate the role of therapeutic treatment in wounds by quantification of epithelialization and granulation tissue formation [30], [31], [32], [33]. In addition, the presence of a cartilage wound base acts to stent open the wound and prevent contraction. In this report, we addressed the effect of topically delivered ASCs in wound repair by quantitative analysis using the rabbit ear model. We characterized rabbit ASCs, and analyzed their multipotency in comparison to BM-MSCs and DFs *in vitro*. In addition, the effect of wound healing by ASCs treatment was compared to BM-MSCs and DFs treatment. Wound analysis suggests that topically delivered ASCs exhibit activated fibroblast phenotype, enhance macrophage recruitment, and increase granulation tissue formation in wounds.

## Materials and Methods

### Isolation and culture of ASCs, BM-MSCs, and DFs

ASCs were isolated as described previously with some modification [11], [31], [34], [35]. Briefly, inguinal fat pads were dissected out from young female New Zealand White rabbits (3–6 months old, ∼2–4 kg) and placed in sterile, pre-warmed phosphate-buffered saline (PBS). Fat pads were then washed several times in PBS, minced manually, and digested in 0.075% collagenase type II in Hank's Buffered Salt Solution (HBSS) for 1 hour at 37°C in a shaking water bath. The stromal vascular fraction (SVF) containing ASCs was isolated by centrifugation at 500× g for 5 minutes, resuspended in HBSS, filtered through a 100 µm sterile nylon mesh filter, and then spun again at 500× g for 5 minutes. The resultant pellet was resuspended in 10 ml of red blood cell (RBC) lysis buffer (10 mM KHCO_3_, 150 mM NH_4_Cl, 0.1 mM EDTA), and allowed to sit at room temperature for 10 minutes. The supernatant was removed by centrifugation following RBC lysis and the pellet was resuspended in Dulbecco's Modified Eagle Medium: Nutrient Mixture F-12 (DMEM/F12) containing 10% fetal bovine serum (FBS, Thermo Scientific, Rockford, IL) and plated in culture dishes. After overnight culture, the media was then removed and replaced with fresh culture medium. The medium was changed twice a week and ASCs were subcultured when they reached 80–90% confluency.

BM-MSCs were isolated from the femoral medullary cavities of rabbits. Bone marrow was collected in PBS containing 2 units/ml heparin and left at room temperature for 10 minutes. After removing the floating fat layer, the solution was added to 5 ml of Ficoll-Paque Plus (1.077 g/ml, GE Healthcare, Piscataway, NJ) and centrifuged at 2,000× g for 30 minutes. The interface layer containing BM-MSCs was recovered and washed in HBSS. BM-MSCs were then cultured in Minimum Essential Medium (MEM) containing 10% FBS.

For the isolation of rabbit DFs, skin tissue was cut into squared pieces (∼1×1 cm^2^) and placed with the epidermis face down in a dish. Dispase (Life Technologies, Carlsbad, CA) with 5 mg/ml in PBS was added and incubated overnight at 4°C. Dermal tissue was minced manually after removing epidermal tissue and digested in 0.25% collagenase type II (Life Technologies) in HBSS at 37°C overnight. The solution was filtered through a 100 µm sterile nylon mesh filter and spun at 500× g for 10 minutes. The pellet was then resuspended in DMEM medium containing 10% FBS and cultured in culture dishes.

### *In vitro* differentiation of MSCs to mesodermal lineage

For adipogenic differentiation, MSCs were seeded in 24 well plates at a concentration of 2×10^4^ and cultured in adipogenesis differentiation medium (Life Technologies). After 8 days culturing, cells were fixed in 4% paraformaldehyde and Oil Red O staining was performed to detect intracellular lipid accumulation. For osteogenic differentiation, MSCs were seeded in collagen (50 µg/ml) coated 24 well plates at a concentration of 1×10^4^ and cultured in osteogenesis differentiation medium (Life Technologies) for 28 or 35 days. Cells were fixed in 4% paraformaldehyde and Alizarin Red S staining was performed to detect accumulated calcium. For chondrogenic differentiation, a total of 8×10^4^ MSCs in 20 µl of culture medium were plated in the middle of 24 well plates. After 3 hours incubation, chondrogenesis differentiation medium was provided (Life Technologies). After 14 or 21 days of culture, cells were fixed in 4% paraformaldehyde. Then, Alcian Blue Staining was performed, which detected sulfated proteoglycan rich matrix.

### Reverse transcription-quantitative PCR (RT-qPCR) and Western blot analysis

Total RNA was prepared by treatment with Trizol Reagent (Sigma-Aldrich, St. Louis, MO) and genomic DNA was removed using the Turbo DNA-free kit (Ambion, Austin, TX). cDNA was made from total RNA using superscript II (Invitrogen, Carlsbad, CA) with random primers. PCR was performed to detect expression of mRNAs. For the quantitative analysis, RT-qPCR analyses using SYBR green I were performed using an ABI prism 7000 sequence detection system (Applied Biosystems, Foster City, CA). Expression of each gene was normalized to the level of glyceraldehyde-3-phosphate dehydrogenase (Gapdh) to get a ΔCt. The 2^−ΔΔCt^ method was used to calculate gene expression difference between differentiated and control samples. Expression of genes was detected by PCR with the following oligonucleotides – Gapdh (5′- AGGTCATCCACGACCACTTC -3′ and 5′- GTGAGTTTCCCGTTCAGCTC -3′), adiponectin (5′- CCTGGTGAGAAGGGTGAAAA -3′ and 5′- GCTGAGCGGTAGACATAGGC -3′), osteopontin (5′- AGGATGAGGACGATGACCAC -3′ and 5′- CACGGCCGTCGTATATTTCT -3′), col10a1 (5′- GGAAAACAAGGGGAGAGAGG -3′ and 5′- CCAGGAGCACCATATCCTGT -3′).

For Western blot analysis, MSCs were washed with PBS, harvested, and lysed with RIPA buffer (150 mM NaCl, 1% NP-40, 0.5% deoxycholic acid, 0.1% SDS, 50 mM Tris-HCl, pH 7.5). Equal amounts of protein were added to a SDS polyacrylamide gel and transblotted on nitrocellulose membranes. Membranes were incubated with anti-CD29 (1∶5,000 dilution; Abcam, Cambridge, MA), anti-CD44 (1∶5,000 dilution; Abcam), anti-CD90 (1∶5,000 dilution; Abcam), or anti-CD105 (1∶2,500 dilution, Abcam) and then incubated with horseradish peroxide-conjugated secondary antibody (1∶5,000 dilution; Vector Laboratories, Burlingame, CA). Specific bands were visualized using an Enhanced Chemiluminescence (ECL) detection kit (GE Healthcare). The blots were probed with anti-β-actin antibody (1∶5,000 dilution; Sigma–Aldrich) to serve as a control for gel loading. The intensity of signal was measured using the NIH image program (ImageJ, http://rsb.info.nih.gov/ij/).

### Labeling of ASCs with green fluorescence protein (GFP)

To stably express GFP, ASCs were transduced with a lentivirus (LV-GFP) in which GFP expression is driven by a cytomegalovirus (CMV) promoter according to the manufacturer's protocol (Life Technologies). Briefly, 2 multiplicity of infection (MOI) of lentivirus was infected to ASCs in the presence of 6 µg/ml of polybrene. Transduced cells were selected by treating 10 µg/ml of blasticidin. GFP expressing cells were further selected by flow cytometry using the Northwestern University Flow Cytometry Facility.

### Treatment of MSCs to the full thickness excisional wounds of rabbit ears

Young, adult New Zealand White rabbits (3–6 months, ∼2–4 kg) were acclimated to standard housing and fed *ad libitum* under an experimental protocol approved by the Northwestern University Animal Care and Use Committee (protocol number: 2010-1841). Rabbits were anesthetized with an intramuscular injection of ketamine and xylazine as described [31], [32]. Wounds were made with a 7 mm surgical punch biopsy (Acuderm, Ft. Lauderdale, FL) down to, but not through, the cartilage. Six wounds were created per ear. Tissue was then elevated in an effort to remove epidermis and dermis, but leave the perichondrium intact. MSCs were topically delivered to wounds in a specific manner to allow each animal to serve as its own internal control; for example, MSCs were delivered into 6 wounds on the one ear and saline was delivered into 6 wounds on the contralateral ear of the rabbits. Wounds are then covered with semi-occlusive dressings (Tegaderm™, 3 M Health Care, St. Paul, MN).Wounds were harvested with a 10 mm surgical punch biopsy tool (Acuderm) at post-operative day (POD) 7 after euthanization with the administration of intracardiac Euthasol followed by a bilateral thoracotomy to assure the death of rabbits. Wounds were immersed in 10% zinc-formalin for fixation.

### Histological and immunochemical analysis of wounds

Formalin-fixed wounds were processed, embedded in paraffin blocks, and then sectioned on a microtome at a thickness of 4 µm. The sections were stained with hematoxylin and eosin (H&E) and histological analysis - epithelial gap and granulation area - was performed using a Nikon Eclipse 50i light microscope and NIS Elements BR software (Nikon, Melville, NY). Slides were analyzed and scored in a blinded fashion and statistical analysis was performed using the Student's *t*-test (two-tailed and unpaired) when comparing 2 study groups, and 1-way analysis of variance (ANOVA) when comparing the means of multiple groups. The level of significance will be set at p<0.05. The n numbers in the histological analysis figures represent total wounds from different rabbits which had 6 wounds per ear.

For the immunostaining, sections were treated with antigen retrieval solution (Dako, Carpinteria, CA) by boiling for 20 minutes before antibody treatment. For immunohistochemistry (IHC), the signal was detected using the Vectastain kit (Vector laboratories) after primary antibody treatment and visualized using 3,3′-diaminobenzidine (DAB). Hematoxylin was used as a counterstain. Mouse anti- alpha smooth muscle actin (α-SMA, 1∶2,000 dilution, Santa Cruz Biotechnology, Santa Cruz, CA), mouse anti- neutrophil Marker (RPN3*/*57, 1∶1,000 dilution, Santa Cruz Biotechnology), mouse anti-CD3 (1∶1,000 dilution, Santa Cruz Biotechnology), and mouse anti-macrophage (1∶1,000 dilution, Abcam) were used as primary antibodies.

For immunofluorescence microscopy, chicken anti-GFP (1∶200 dilution, Life Technologies), α-SMA (1∶200 dilution, Santa Cruz Biotechnology), mouse anti-collagen III (col3, 1∶200 dilution, Novus Biologicals, Littleton, CO), mouse anti-CD31 (1∶25 dilution, Abcam), mouse anti-Ki67 (1∶20 dilution, Novocastra, Buffalo Grove, IL), and mouse anti-PCNA (1∶100 dilution, BD Biosciences, San Jose, CA) antibodies were used as primary antibodies. Alexa Fluor 488 or 555 conjugated secondary antibodies were used to detect the primary antibody (Invitrogen). Nuclei were stained with 4′,6-diamidino-2-phenylindole (DAPI, 1 µg/ml).

## Results

### Isolation and characterization of rabbit ASCs

**Figure 1:**
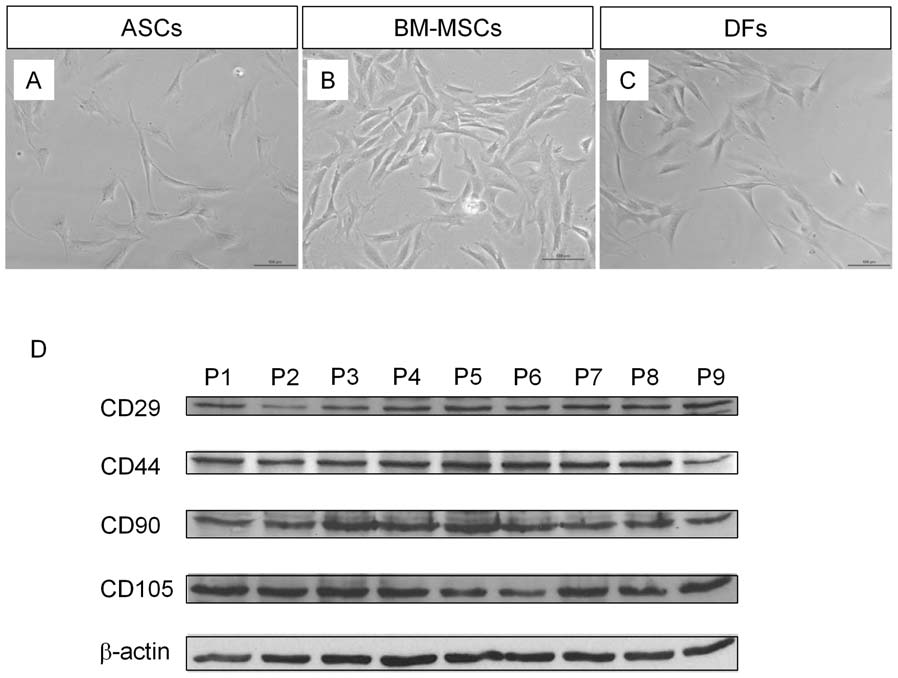
Morphology and surface markers of rabbit MSCs. (A–C): Bright field images of rabbit ASCs (A), BM-MSCs (B), and DFs (C). Cells were grown in culture dishes with growth medium and photos were taken. Scale bar; 100 µm. (D): Western blot analysis. Whole cell extract of rabbit ASCs from passage 1 (P1) to P9 was prepared and loaded 20 µg per well. The expression of CD29, CD44, CD90, and CD105 were detected with their specific antibodies as indicated. β-actin was detected as a loading control.

Rabbit ASCs have spindle shapes during *in vitro* primary culture and are morphologically similar to DFs (Figure 1A & 1C). Rabbit BM-MSCs have a larger surface area compared to ASCs (Figure 1B). We characterized ASCs by analyzing surface markers and multipotency of differentiation. Unlike embryonic stem cells, which have specific makers such as Oct-4 and SSEA, MSCs cannot be characterized by specific markers because definitive cellular markers are not yet identified. Thus, a series of positive and negative surface markers are needed for the characterization of MSCs [7], [9], [13], [15], [16], [25], [36]. We selected CD29, CD44, CD 90, and CD105 as positive markers. Two hematopoietic cell markers, CD34 and CD45, were used as negative markers. Given the limited information of antibodies in rabbit protein, we tested antibodies that were designed to detect human antigens. Specificity of antibodies, except CD45, was confirmed by Western blot analysis (data not shown). Expression of CD34 was detected in neither ASCs nor BM-MSCs (data not shown). We tested antibodies from four different vendors but could not find antibodies which are specific to rabbit CD45 protein (data not shown). Expression of CD29, CD44, CD90, and CD105 was detected without significant changes, though minor variations were found when quantified with the NIH ImageJ program, from passage 1 through passage 9 both in ASCs (Figure 1D) and BM-MSCs (Figure S1).

### Multi-lineage differentiation potential of ASCs

**Figure 2:**
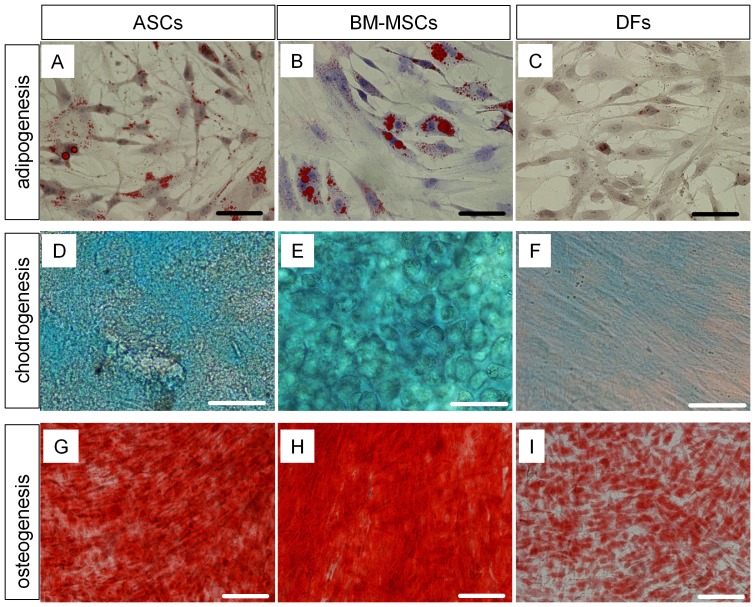
Rabbit MSCs differentiate to mesodermal lineages *in vitro*. Passage 2 ASCs (A, D, G), BM-MSCs (B, E, H), and DFs (C, F, I) were used for differentiation. (A–C): Adipogenic differentiation. Cells were cultured in adipogenesis differentiation medium for 8 days. Oil Red O staining was performed to detect lipid accumulation. Nuclei were stained with Hematoxylin. (D–F): Chondrogenic differentiation. Cells were cultured in chondrogenesis differentiation medium for 21 days. Alcian blue staining was performed. (G–I): Osteogenic differentiation. Cells were cultured in osteogenesis differentiation medium for 35 days. Alizarin Red S staining was performed to detect calcium accumulation. Abbreviation: MSCs, mesenchymal stem cells; ASCs, adipose derived stem cells; DFs, dermal fibroblasts; BM-MSCs, bone marrow derived mesenchymal stem cell. Scale bar; (A–F) 50 µm, (G–I) 100 µm.

We addressed the multipotency of ASCs, and compared them to BM-MSCs and DFs. For adipogeneis, Oil Red O staining showed an accumulation of lipid droplets in the cytoplasm of ASCs and BM-MSCs which were grown in adipogenesis medium for 8 days (Figure 2A & B). In contrast, fewer lipid droplets were found in the cytoplasm of DFs (Figure 2C). Alcian Blue staining showed positive signals in ASCs and BM-MSCs which were cultured in chondrogensis medium for 14 days and signals were strengthened at day 21 culture (Figure 2D & E). Alcian Blue staining positive signals were found in DFs culture, though those signals were weaker compared to ASCs and BM-MSCs (Figure 2F). Accumulated calcium was detected in ASCs and BM-MSCs but not in DFs by Alizarin Red S staining at day 28 culture in the osteogenesis medium (data not shown). Both ASCs and BM-MSCs showed a high accumulation of calcium at day 35 culture (Figure 2G & H). DFs also showed an accumulation of calcium, although it was a smaller amount compared to ASCs and BM-MSCs in the day 35 culture (Figure 2I).

We analyzed the expression of specific genes of adipocyte, osteocyte, and chondrocyte lineages by RT-qPCR [11]. Expression of an adipocyte specific gene, adiponectin, was increased by 35-fold and 17-fold in ASCs and BM-MSCs, respectively, when cultured in adipogenic medium for 8 days (Figure S2A). However, induction of adiponectin in DFs was not found in the same culture condition. Expression of an osteocyte specific gene, osteopontin, was increased by 3.2-fold and 2.7-fold in ASCs and BM-MSCs, respectively. This increase in gene expression was not found in DFs when it was cultured in osteogenic medium for 28 days (Figure S2B). Expression of a chondrocyte specific gene, Col10a1, was increased by 6-fold, 12-fold, and 1,515-fold in DFs, ASCs, and BM-MSCs, respectively, when cultured in chondrogenic medium for 21 days (Figure S2C). These results suggest that ASCs and BM-MSCs have differential properties, though they share similar surface markers and have multilineage differentiation potential. ASCs and BM-MSCs are prone to differentiate into adipocytes and chondrocytes, respectively. DFs have less multipotency compared to ASCs and BM-MSCs.

### ASCs enhance granulation tissue formation in wounds

To determine the optimal quantity of ASCs to promote wound healing, we treated wounds with different amounts of ASCs. Though we showed the conservation of surface markers of ASCs *in vitro*, we used an early passage (P3) ASCs in these experiments to avoid changes of characteristics of ASCs over the long term *in vitro* culture. ASCs were harvested, washed in PBS to remove cell culture medium, and resuspended in PBS. Three different amounts of ASCs - 3×10^5^, 1×10^5^, and 3×10^4^ - in 7 µl of PBS were delivered to each 7 mm wound of one ear. In the contralateral ear, 7 µl of PBS were delivered to each wound as a control. Wounds were harvested at POD7 and histological differences such as epithelial gap and granulation tissue areas were digitally quantified as previously described (Figure S3) [30], [31]. All three concentrations of ASCs increased granulation tissue area (data not shown). Wounds treated with highest ASCs number - 3×10^5^ - had a larger epithelial gap. This means there was minor inhibition of keratinocyte migration, though this was not statistically significant (data not shown).

To determine the dose of ASCs which does not inhibit epithelialization but increases granulation tissue formation, wounds on one ear were treated with 1×10^5^ ASCs and wounds on the contralateral ear were treated with 3×10^4^ ASCs. Wounds with 1×10^5^ ASCs (n = 11) had similar epithelial gaps (4.35+0.42 mm, Figure S4A) compared to wounds with 3×10^4^ ASCs (n = 12, 4.09+0.32 mm). However, wounds treated with 1×10^5^ ASCs showed greater granulation tissue area, though it did not reach statistically significance (Figure S4B, 1.48±0.21 vs. 0.95±0.13 mm**^2^**, p = 0.09). Thus we determined that 1×10^5^ ASCs as an optimum number for wound healing study.

**Figure 3:**
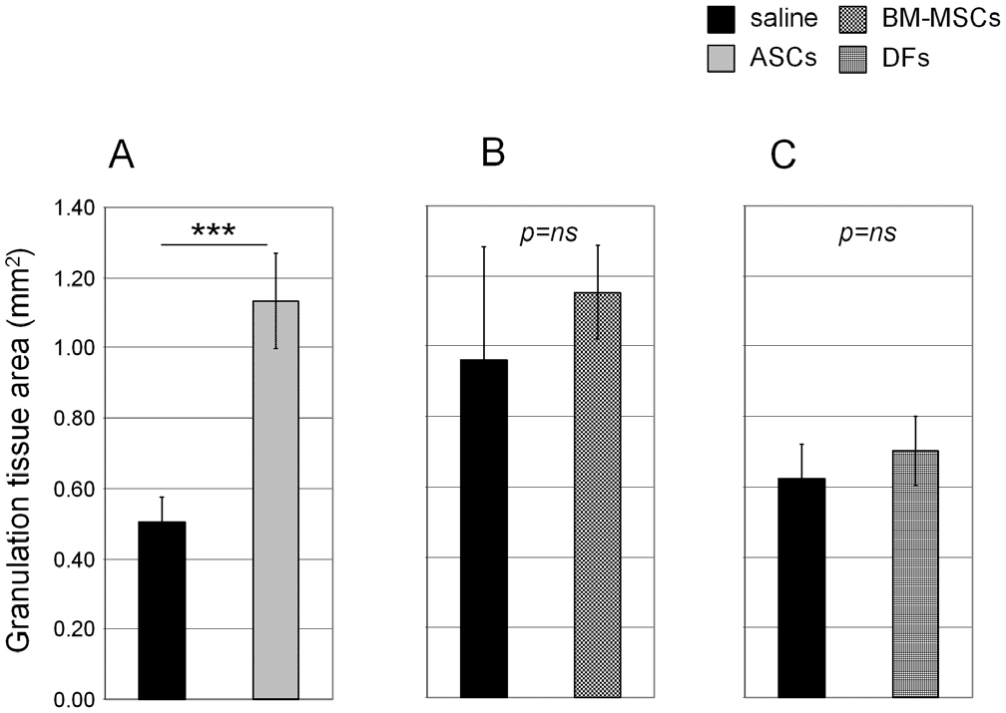
Histological quantification of MSCs treated wounds. (A–C): A total of 1×10^5^ ASCs (A), BM-MSCs (B), and DFs (C) in PBS were delivered to 7 mm wounds on one ear. In the contralateral ear, PBS alone was delivered as a control. Wounds were harvested at POD7 and granulation tissue area was measured. Number of wounds analyzed; (A, n = 35 for saline & n = 36 for ASCs; B, n = 17 for saline & n = 20 for BM-MSCs, C, n = 17 for saline & n = 24 for DFs). N represents the total number of wounds from six (A) or four (B, C) rabbits. Data shown as mean + SEM. ***p<0.001, ns = not significant.

Next, we increased the number of wounds and animals to increase power of statistical analyses. With each rabbit serving as its own internal control, 1×10^5^ of P3 ASCs were delivered to wounds on one ear and the wounds on contralateral ear received saline alone as control as described above and wounds were analyzed at POD7. When compared to saline treated control wounds, ASCs treated wounds showed increased granulation tissue area (0.50±0.07 mm**^2^** vs. 1.13±0.14 mm**^2^**, p = 0.0001, Figure 3A). ASCs treatment did not affect epithelialization of the epidermis when compared to saline treated wounds (4.03±0.31 mm vs. 3.91±0.26 mm, respectively; p = 0.8, Figure S5). For comparison, 1×10^5^ of P3 BM-MSCs (or DFs) were delivered to wounds on one ear and saline control were delivered to wounds on the contralateral ear. We found that granulation tissue area was not significantly changed by BM-MSCs (0.96+0.32 mm**^2^** vs. 1.15+0.13 mm**^2^**, Figure 3B) or DFs (0.62±0.10 mm**^2^** vs. 0.70±0.10 mm**^2^**, Figure 3C) treatment when compared to saline treated wounds. We also performed an ANOVA test to compare the effect of three different cell types - ASCs, BM-MSCs, and DFs, on wound repair (Figure S6). The post hoc *t*-test showed the significant difference between the ASCs and DFs, BM-MSCs and DFs, while there's no statistically significant difference between ASCs and BM-MSCs. However, given the variation among rabbits which are not syngeneic, each rabbit served as its own internal control in our wound healing analyses. Thus, we think that the Student *t*-test is more accurate than the ANOVA test for our purpose.

### Transplanted ASCs exhibit activated fibroblast phenotype

**Figure 4:**
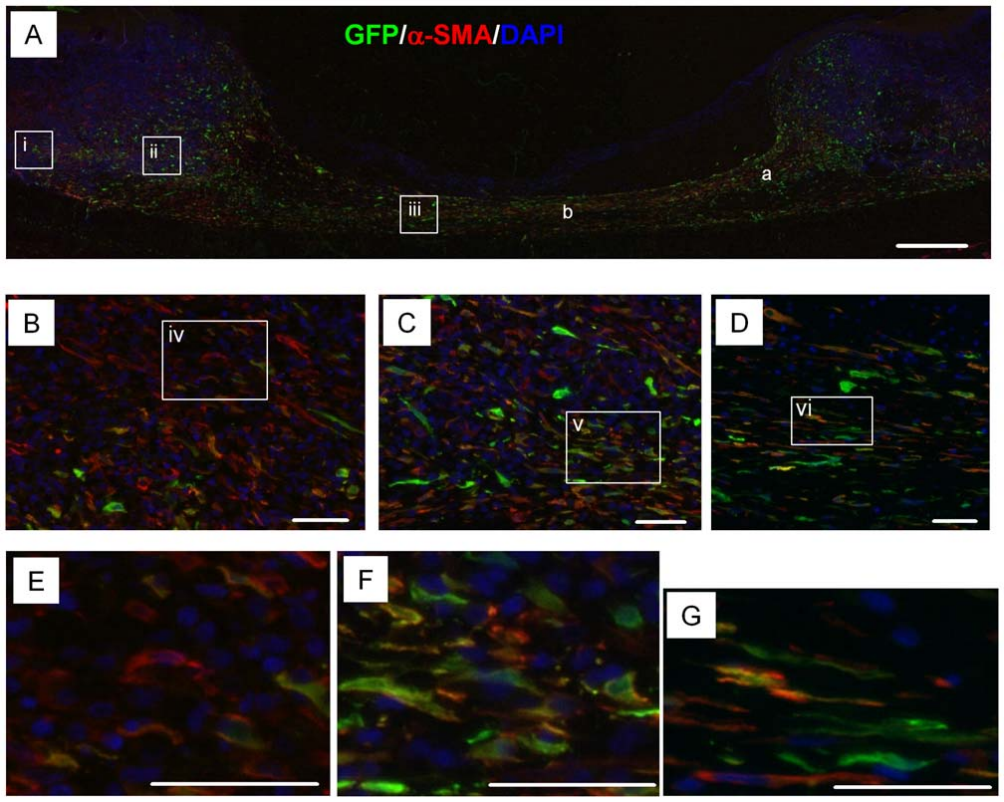
Transplanted ASCs express α-SMA in wounds. GFP-expressing ASCs were analyzed 7 days after transplantation in wounds. Chicken anti-GFP and mouse anti-α-SMA antibodies were used to detect GFP and α-SMA. Nuclei were stained with DAPI. (A): Low magnification of wounds. The areas analyzed in Figure 6 were indicated by ‘a’ and ‘b’. (B–D): Higher magnifications of the indicated regions in A (white squares; labeled as i, ii, iii). (E–G): Higher magnifications of the indicated regions in B–D (white squares; labeled as iv, v, vi). Merged images of α-SMA (red) and GFP (green) indicate that α-SMA is expressed in ASCs. Scale bars: 500 µm (A), 50 µm (B–G).

Wound healing is a complex process in which interactions of diverse cell types and cytokines are involved. ASCs can contribute to wound healing by either cytokine expression, or differentiation and repopulation in wounds. We analyzed the transplanted ASCs in wounds using GFP-expressing ASCs (GFP-ASCs). A total of 1×10^5^ GFP-ASCs in saline was delivered into each wound. Wounds were harvested at POD7 and histological analysis was performed. Immunofluorescence staining with anti-GFP antibody showed that transplanted ASCs were evenly distributed in the wound bed and granulation area (Figure 4A & Figure S8A). During the wound repair process, fibroblasts migrate to the wound site and build up granulation tissue by depositing collagen and other extracellular matrices [37], [38]. These activated myofibroblasts are characterized by α-SMA expression. Immunofluorescence staining with anti-α-SMA antibody detected endogenously activated fibroblasts (cells with red color, Figure 4 & Figure S7). Interestingly, the majority of transplanted ASCs showed α-SMA signal (cells with yellow color, Figure 4C, D, F, G & Figure S7). We also observed ASCs which did not express α-SMA (cells with green color, Figure 4 & Figure S7).

**Figure 5:**
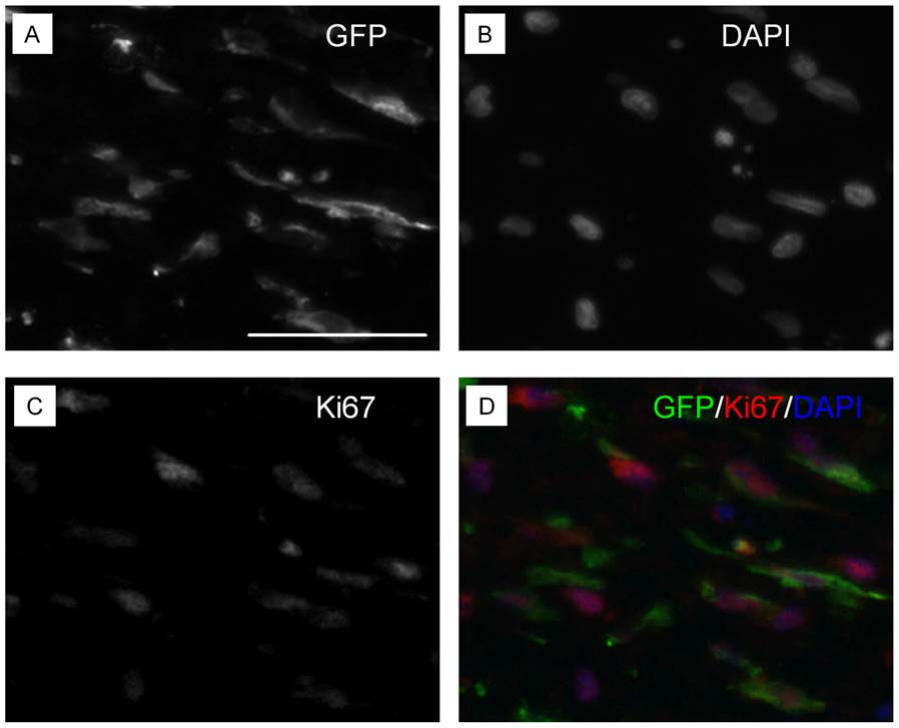
Transplanted ASCs proliferate in wounds. GFP-expressing ASCs were analyzed 7 days after transplantation in wounds. Chicken anti-GFP (A) and mouse anti-Ki67 (C) antibodies were used. Nuclei were stained with DAPI (B). Merged image was shown in D. Scale bars: 50 µm.

We next analyzed expression of collagen III (Col III), which is produced by myofibroblasts before synthesis of mechanically stronger collagen I. Expression of Col III was detected in wound bed (Figure S8A, C, E) and granulation tissue (Figure S8A, D, F), though the signal was weak. Expression of Col III was detected in the outside of wounded area (Figure S8A & B). Transdifferentiation of ASCs to endothelial cells was addressed using a platelet endothelial cell adhesion molecule (PECAM-1, CD31) - specific antibody. Expression of CD31 was prominently detected in the granulation tissue (Figure S9). However, co-expression of CD31 in transplanted ASCs was not detected (Figure S9). Thus, transdifferentiation of transplanted ASCs to endothelial cell was not found at POD7 in the rabbit wounds. Proliferation of transplanted ASCs was detected with Ki-67 or PCNA specific antibodies (Figure 5 & Figure S10).

### Analysis of cells involved in wound healing in the ASCs treated wounds

**Figure 6:**
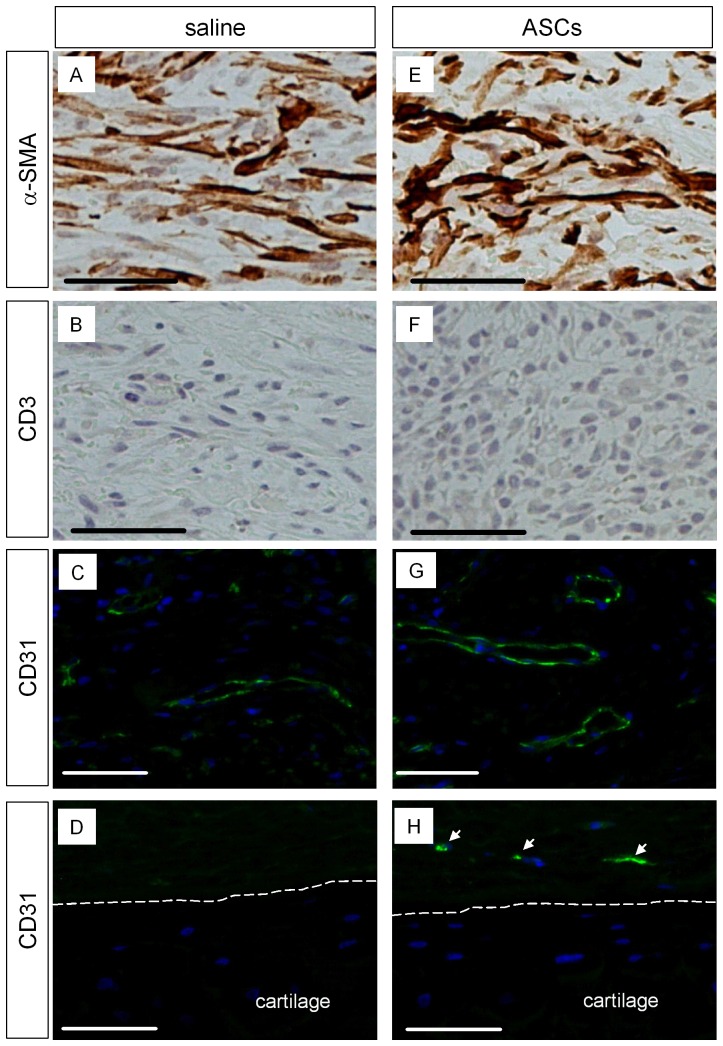
Analysis of protein expression in ASCs treated wounds. Saline control (A, B, C, D) and ASCs (E, F, G, H) were delivered to wounds and harvested as described in Figure 3. α-SMA (A, E) and CD3 (B, F) were visualized by DAB after staining with their specific antibodies. CD31 (C, D, G, H) was stained with its specific antibody and visualized using fluorescence conjugated secondary antibody. (A, B, C, E, F, G): images were taken from the area labeled as ‘a’ in Figure 4. (D, H): Immunostaining for CD31 in the area labeled as ‘b’ in Figure 4. The junction area between cartilage and wound beds was demarcated by white dot lines. CD31 positive signals were indicated by arrows in H. Scale bars: 50 µm.

We further analyzed ASCs treated wounds immunohistochemically and compared them with saline treated wounds. Expression of α-SMA was found in the granulation tissue of ASCs treated wounds and saline treated control wounds (Figure 6A, E). α-SMA signals in ASCs treated wounds (Figure 6E) are from endogenous activated fibroblast cells and transplanted ASCs (Figure 4 & Figure S7). α-SMA signals in Figure 6A are from endogenous activated fibroblast cells. The transplanted ASCs are allogeneic because syngeneic rabbits are not available. We investigated whether transplanted allogeneic ASCs evoke immune reactions *in vivo*, though immune modulatory property of ASCs has been proposed [16], [20], [21], [39]. Neither CD3 (T cell antigen) nor CD45 (common leukocyte antigen) positive signals were found by their specific antibodies in ASCs treated wounds at POD7 (Figure 6F & data not shown). Angiogenesis is one of critical factors in wound repair process. Blood vessel formation in granulation tissue, which is determined by endothelial marker (CD31) staining, was detected in ASCs treated wounds and control wounds (Figure 6C, G). Interestingly, a few CD31 positive cells were found in the wound beds of ASCs treated wounds, though blood vessel structure was not found (Figure 6H). In contrast, we could not detect CD31 positive cells in the wound beds of saline treated wounds at POD7 (Figure 6D). Since transdifferentiation of ASC to endothelial cells was not found at POD7 in our animal model (Figure S9), we suggest that ASCs increase endothelial cell recruitment in wounds.

**Figure 7:**
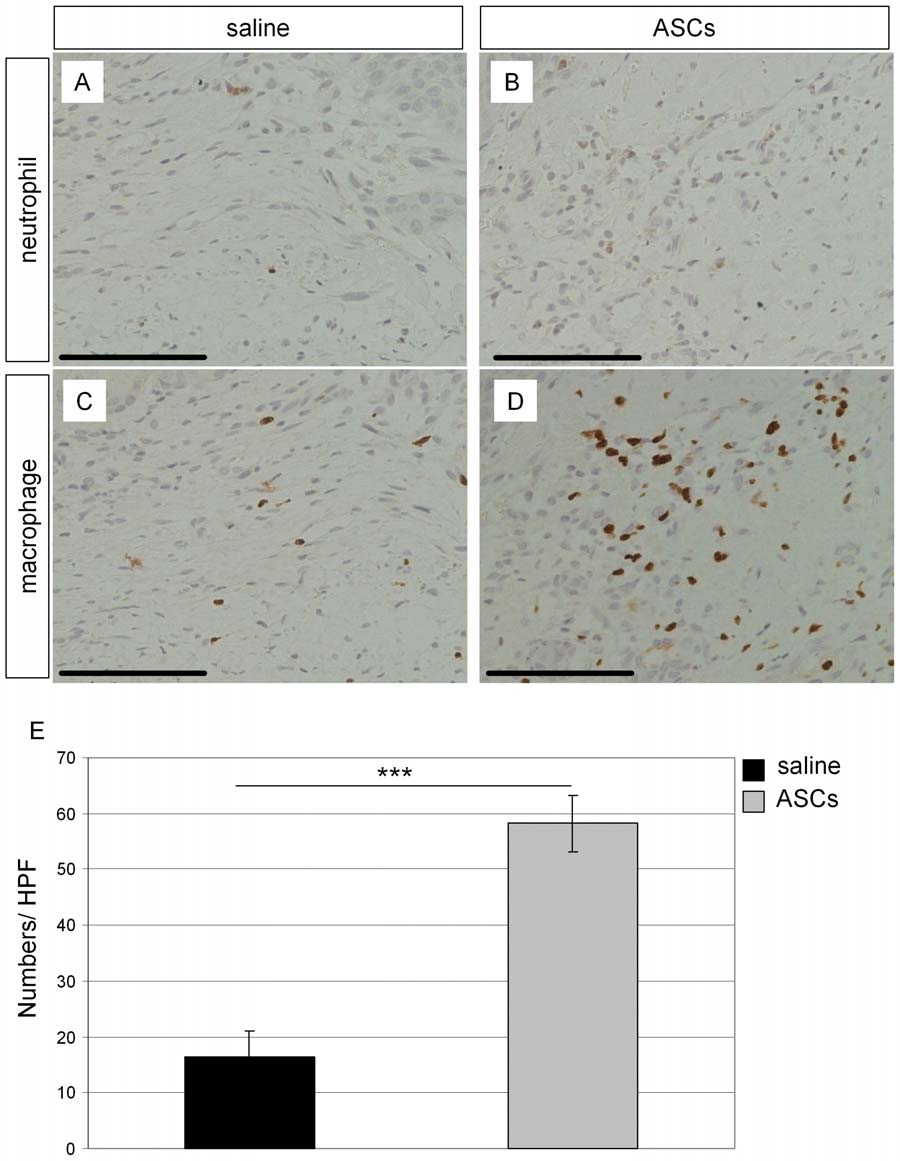
Higher infiltration of macrophages was found in ASCs treated wounds. Saline control (A, C) and ASCs (B, D) were delivered to wounds and harvested as described in Figure 3. Neutrophils (A, B) and macrophages (C, D) were visualized by DAB after staining with their specific antibodies. Scale bars: 100 µm. (E): Number of macrophages per high-power microscopic fields (HPF) at 400 X magnification. Macrophages were counted and averaged from four HPF. Data are from four independent wounds and presented as mean + SEM. ***p<0.001.

Neutrophils migrate to wounded sites upon injury and initiate an initial inflammatory phase for wound repair. Then, macrophages move to the sites and secrete cytokines and growth factors which attract cells involved in wound repair [40], [41]. They participate in remodeling the extracellular matrix and forming granulation tissue to repair wounds. We did not detect a significant number of neutrophils at POD7 wounds in which ASCs or saline controls were treated (Figure 7A, B). We detected average of 16.4 macrophages in the granulation tissue near to the migrating epidermis with high power microscopic fields (HPF, Figure 7C, E). The number of macrophages in the granulation tissue was markedly increased by ASCs treatment at POD7 (Figure 7D, E). Thus, our wound analyses (through Figures 3 to 7) suggest that transplanted ASCs exhibit the activated fibroblast phenotype, increase endothelial cell recruitment, and enhance wound repair by macrophage recruitment.

## Discussion

BM-MSCs were first isolated among various MSCs and have potential to contribute to wound repair in many tissues. However, the procedure for extracting BM-MSCs is relatively invasive and could cause patient morbidity. Extended time is required to expand BM-MSCs in culture to get a large enough number of cells for clinical uses. ASCs have several advantages compared with BM-MSCs because they are easy to isolate with relative abundance [9], [14], [27]. ASCs and BM-MSCs have similar surface markers, cytokines and gene expression profiles [7], [9], [16], [42]. Thus, we selected ASCs as a cell therapy reagent for wound repair in this report and compared their properties to BM-MSCs. The mesodermal lineage differentiation experiment suggests that ASCs are more likely to differentiate into adipocytes, while BM-MSCs are prone to differentiate into chondrocytes (Figure 2). ASCs engrafts enhanced granulation tissue area in rabbit ear wounds, while BM-MSCs engrafts did not increase granulation tissue area (Figure 3). It is known that granulation tissue formation is not always beneficial because prolonged activation of fibroblasts in dermis increases granulation tissue formation and results in hypertrophic scar [1], [43], [44]. However, it is not easy to tell whether the granulation tissue is healthy or not in early phase, the time window we analyzed, because formation of granulation tissue is essential during the wound healing process. We have done initial experiments analyzing the effects of MSCs on scarring and found no evidence of increased scarring with MSCs in spite of their increased cellularity in the wound healing experiments. However, in an analysis of scar formation at 28 days, there was no significant effect on scar area or elevation index (data not shown). Further investigation is needed to regenerate, not repair, tissue with MSCs treatment; for example, testing different conditions such as using matrices as delivery vehicles or initially growing the cells in 3-dimensional cultures which may optimize their properties [1], [26], [45], [46].

Our results further support the finding suggesting ASCs and BM-MSCs are not identical, though they have similar surface markers [47], [48], [49], [50]. DFs are poorly characterized diverse cells which locate in dermis. Upon injury DFs are activated and become myofibroblasts, which express α-SMA and cytokines while depositing extracellular matrix [51]. It has been shown that human ASCs and DFs display similar surface markers and multipotency to differentiate into osteocytes, adipocytes, and chondrocytes [52], [53], [54], [55]. They have similar chemokine expression profiles. However despite the morphological similarity, they are not identical [56], [57]. Our analysis showed that DFs have less potency to be differentiated compared to ASCs and BM-MSCs (Figure 2). While the use of DFs in wound repair has been reported [31], [58], the DFs engraft did not enhance granulation tissue area in rabbit ear wounds (Figure 3).

Even though ASCs are a promising candidate for cell therapy, there are several pitfalls to be addressed. First, it is expected that engrafted ASCs participate in wound repair by either paracrine signaling or direct differentiation to specific cell types such as endothelial cells or keratinocytes. There are reports which showed transdifferentiation of ASCs *in vivo* [26], [59]. However, it is speculated that the major role of engrafted ASCs is secreting cytokines and growth factors, which enhance wound repair (see the following paragraph). Multipotency of ASCs has been tested *in vitro* where signals are provided to differentiate ASCs to specific cell types; however, this is unlikely to happen *in vivo*. Therefore, further investigation to find the optimum microenvironment for ASCs differentiation is essential in the complex and multicellular process of wound repair. Second, allogeneic and xenogeneic therapeutics have been considered because they have reduced expression of histocompatibility antigens and secrete immunoregulatory molecules [10], [15], [16]. However, investigation of immune reaction by comparing autologous ASCs in quantitative analysis is needed. Third, a limiting factor of ASCs is that they differ in proliferation and differentiation capacity depending on age, gender, and the location in the body from which the cells are derived [41], [42], [43]. Understanding the underlining mechanisms, such as epigenetic control, is needed for the clinical use of non-autologous ASCs.

Though there are disputes on transdifferentiation of ASCs *in vivo*, costaining of engrafted ASCs with other cell types has been reported *in vivo*. ASCs are differentiated to endothelial and epidermal cells in the murine model 2–4 weeks after delivery [26]. However, costaining of ASCs with endothelial marker was not found in the rabbit wounds 7 days after delivery (Figure S9). Therefore, we suspect that 7 days are not enough for rabbit ASCs to be transdifferentiated to other cells or that the microenvironment in rabbit wounds is different from that in murine wounds. There are reports which suggest that engrafted ASCs enhance angiogenesis by releasing angiogenic factors [22], [60], [61], [62]. In line with this, we found increased CD31 positive cells in wound bed in ASCs treated wounds (Figure 6H), though direct differentiation of ASCs to endothelial cells was not detected. It has been suggested that MSCs contribute to tissue repair via secretion of soluble factors rather than transdifferentiation [7], [63]. We anticipate that the paracrine effect of ASCs in wound healing is crucial for the healing of wounds in which large volume of tissues is lost.

Macrophages play key roles during the wound repair process which includes inflammation, granulation formation, and remodeling in wounds [40]. It has been shown that macrophages promote wound repair after skin injury [64], [65], [66], [67]. Myofibroblasts are activated fibroblasts and express α-SMA. They play a critical role in wound repair by depositing extracellular matrices such as fibronectin and collagen, and by secreting proteinases which remodel the matrix [68]. Thus, both myofibroblasts and macrophages are critical players in wound repair. Engrafted ASCs showed the myofibroblast phenotype in rabbit ear wound (Figure 4). In addition, the number of infiltrated macrophages was increased in ASCs treated wounds (Figure 7). These data suggest that transplanted ASCs enhance granulation tissue formation via their activated fibroblast phenotype and increased recruitment of macrophages in wounds.

## Conclusions

ASCs have advantages as cell therapy agents as compared with other MSCs, such as BM-MSCs. This is because they are easy to isolate with relative abundance. We confirmed that MSCs surface markers (CD29, CD44, CD 90, and CD105) are expressed in rabbit ASCs and are maintained in *in vitro* culture. Rabbit ASCs have the ability to differentiate into mesodermal cells such as adipocytes, chondrocytes, and osteocytes. Topically delivered ASCs proliferated in the wounds exhibit the activated fibroblast phenotype. Engrafted ASCs increased macrophage recruitment and enhanced granulation tissue formation in wounds. Our data support ASCs as a possible cell therapy candidate for the repair of wounds.

## Supporting Information

Figure S1 **Western blot analysis for surface markers of rabbit BM-MSCs.** Whole cell extract of rabbit BM-MSCs from P1 to P9 was prepared and loaded 20 µg per well. The expression of CD29, CD44, CD90, and CD105 were detected with their specific antibodies as indicated. β-actin was detected as a loading control.
(PDF)

Figure S2 **mRNA level of lineage specific genes was increased by differentiation in MSCs.** DFs, ASCs, and BM-MSCs were grown in adipogenic (A), osteogenic (B), or chondrogenic (C) medium for 8, 28, or 21 days. Total RNAs were isolated and RT-qPCR was performed. Expression of adiponectin (A), osteopontin (B), and Col10a1 (C) was analyzed. Each gene expression was normalized according to the expression level of Gapdh. Data are from a single representative experiment. The level of gene expression in cells cultured in differentiation medium was compared to cells cultured in non-differentiated medium, which was set at 1.
(PDF)

Figure S3 **Schematic drawing of rabbit wounds and histological analysis.** EG, epithelial gap; GA, granulation area.
(PDF)

Figure S4 **Histological quantification of ASCs treated wounds.** 1×10^5^ ASCs were delivered to 7 mm wounds on one ear and 3×10^4^ ASCs were delivered to wounds on the contralateral ear of rabbits. Wounds were harvested at POD7 and epithelial gap (A) and granulation tissue area (B) were measured (n = 11 for 1×10^5^ ASCs & n = 12 for 3×10^4^ ASCs). N represents the total number of wounds from two rabbits. Data shown as mean + SEM. ns = not significant.
(PDF)

Figure S5 **Measurement of epithelial gap of ASCs treated wounds.** A total of 1×10^5^ ASCs were delivered to 7 mm wounds on one ear. In the contralateral ear, PBS alone was delivered as a control. Wounds were harvested at POD7 and epithelial gap was measured. Data shown as mean + SEM. n = 35 for saline & n = 36 for ASCs. N represents the total number of wounds from six rabbits. ns = not significant.
(PDF)

Figure S6 **Comparison of the effect of ASCs, BM-MSCs, and DFs on wound repair.** The granulation tissue measurement data at POD7 wounds in Figure 3 was re-analyzed by the ANOVA with post-hoc analysis. Number of wounds analyzed; n = 69 for saline, n = 20 for BM-MSCs, n = 36 for ASCs, n = 24 for DFs. N represents the total number of wounds from fourteen (saline), six (ASCs), or four (DFs & DM-MSCs) rabbits. Data shown as mean + SEM. *p<0.05, **p<0.01.
(PDF)

Figure S7 **Analysis of α-SMA expressing cells in wounds.** Chicken anti-GFP and mouse anti-α-SMA antibodies were used to detect GFP and α-SMA. Nuclei were stained with DAPI. GFP (A, D, G), α-SMA (B, E, H), and merged (C, F, I) images in Figure 4E, 4F, 4G were shown. Endogenous cells and transplanted ASCs which express α-SMA showed red and yellow color, respectively, in the merged images (C, F, I). Transplanted ASCs which do not express α-SMA showed green color in the merged images. Scale bars: 50 µm.
(PDF)

Figure S8 **Expression of collagen III (Col III) in wounds.** GFP-expressing ASCs were analyzed 7 days after transplantation in wounds. Chicken anti-GFP and mouse anti-Col III antibodies were used to detect GFP (green) and Col III (red). Nuclei were stained with DAPI. (A): Low magnification of wounds. (B-D): Higher magnifications of the indicated regions in A (white squares; labeled as i, ii, iii). (E–F): Higher magnifications of the indicated regions in C and D (white squares; labeled as iv and v). Merged images of Col III and GFP were shown. Scale bars: 500 µm (A), 100 µm (B, C, D), 50 µm (E, F).
(PDF)

Figure S9 **Analysis of expression of CD31 (PECAM-1) in transplanted ASCs.** GFP-expressing ASCs were analyzed 7 days after transplantation in wounds. Chicken anti-GFP (A, D) and mouse anti-CD31 (B, E) antibodies were used to detect GFP and CD31. Nuclei were stained with DAPI. Co-expression of CD31 and GFP was not detected in the merged images (C, F). Two examples, section 1 and section 2, from the same wound were shown. Scale bar; 50 µm.
(PDF)

Figure S10 **Transplanted ASCs proliferate in wounds.** GFP-expressing ASCs were analyzed 7 days after transplantation in wounds. Chicken anti-GFP (A) and mouse anti-PCNA (C) antibodies were used. Nuclei were stained with DAPI (B). Merged image was shown in D. Scale bars: 50 µm.
(PDF)
