# Supplementary figures and images for: Topically Delivered Adipose Derived Stem Cells Show an Activated-Fibroblast Phenotype and Enhance Granulation Tissue Formation in Skin Wounds
Source: PLoS One. 2013 Jan 31;8(1):e55640. doi: 10.1371/journal.pone.0055640 (PMC3561304; doi:10.1371/journal.pone.0055640)

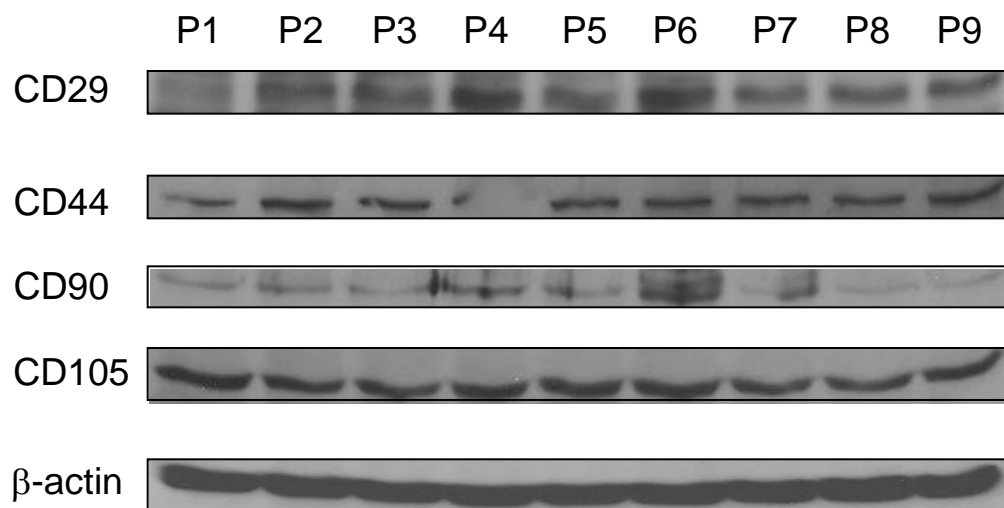

Supplemental Figure S1

Supplement: Figure S1 — Western blot analysis for surface markers of rabbit BM-MSCs. Whole cell extract of rabbit BM-MSCs from P1 to P9 was prepared and loaded 20 µg per well. The expression of CD29, CD44, CD90, and CD105 were detected with their specific antibodies as indicated. β-actin was detected as a loading control. (PDF) [file pone.0055640.s001.pdf]

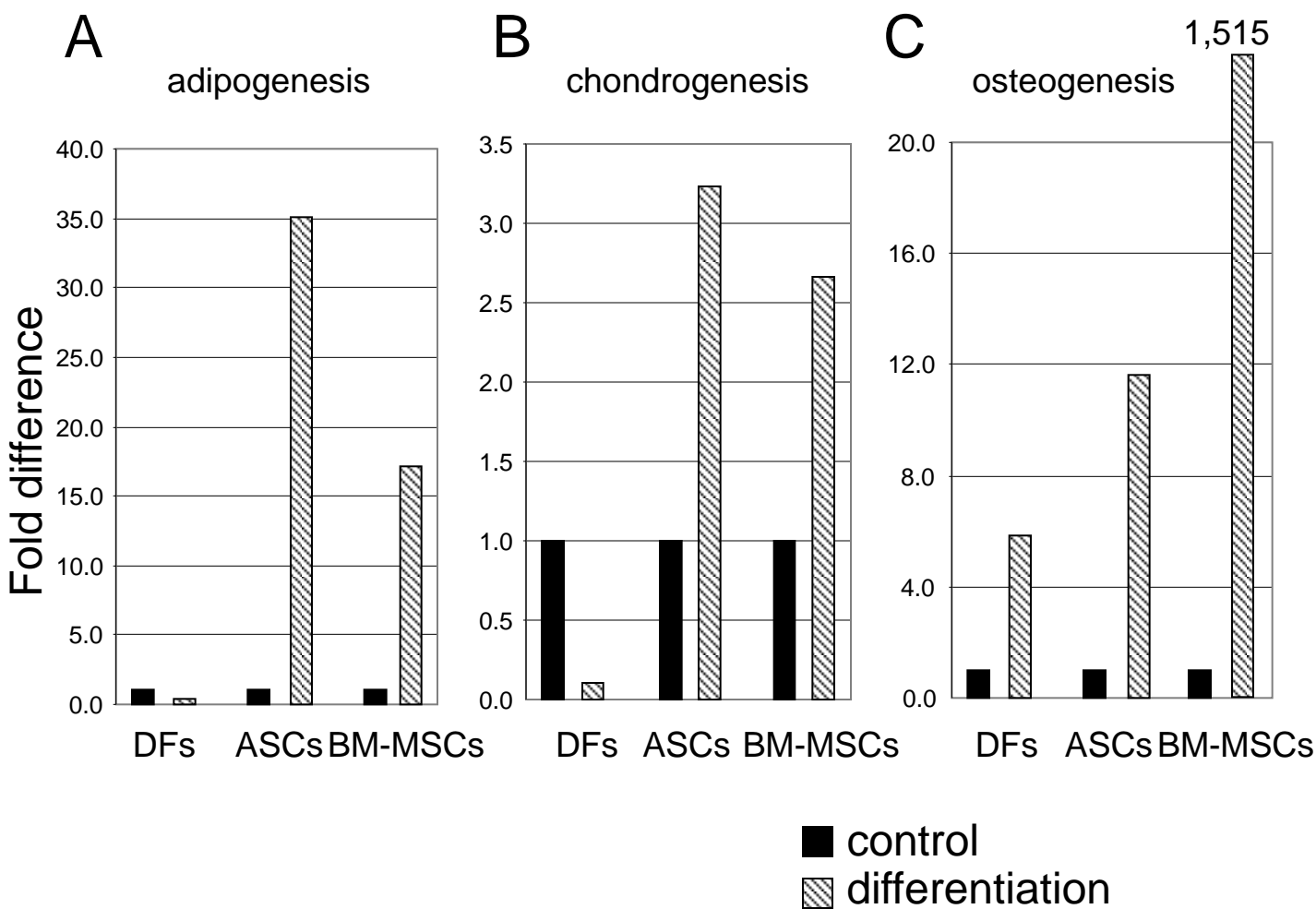

Supplemental Figure S2

Supplement: Figure S2 — mRNA level of lineage specific genes was increased by differentiation in MSCs. DFs, ASCs, and BM-MSCs were grown in adipogenic (A), osteogenic (B), or chondrogenic (C) medium for 8, 28, or 21 days. Total RNAs were isolated and RT-qPCR was performed. Expression of adiponectin (A), osteopontin (B), and Col10a1 (C) was analyzed. Each gene expression was normalized according to the expression level of Gapdh. Data are from a single representative experiment. The level of gene expression in cells cultured in differentiation medium was compared to cells cultured in non-differentiated medium, which was set at 1. (PDF) [file pone.0055640.s002.pdf]

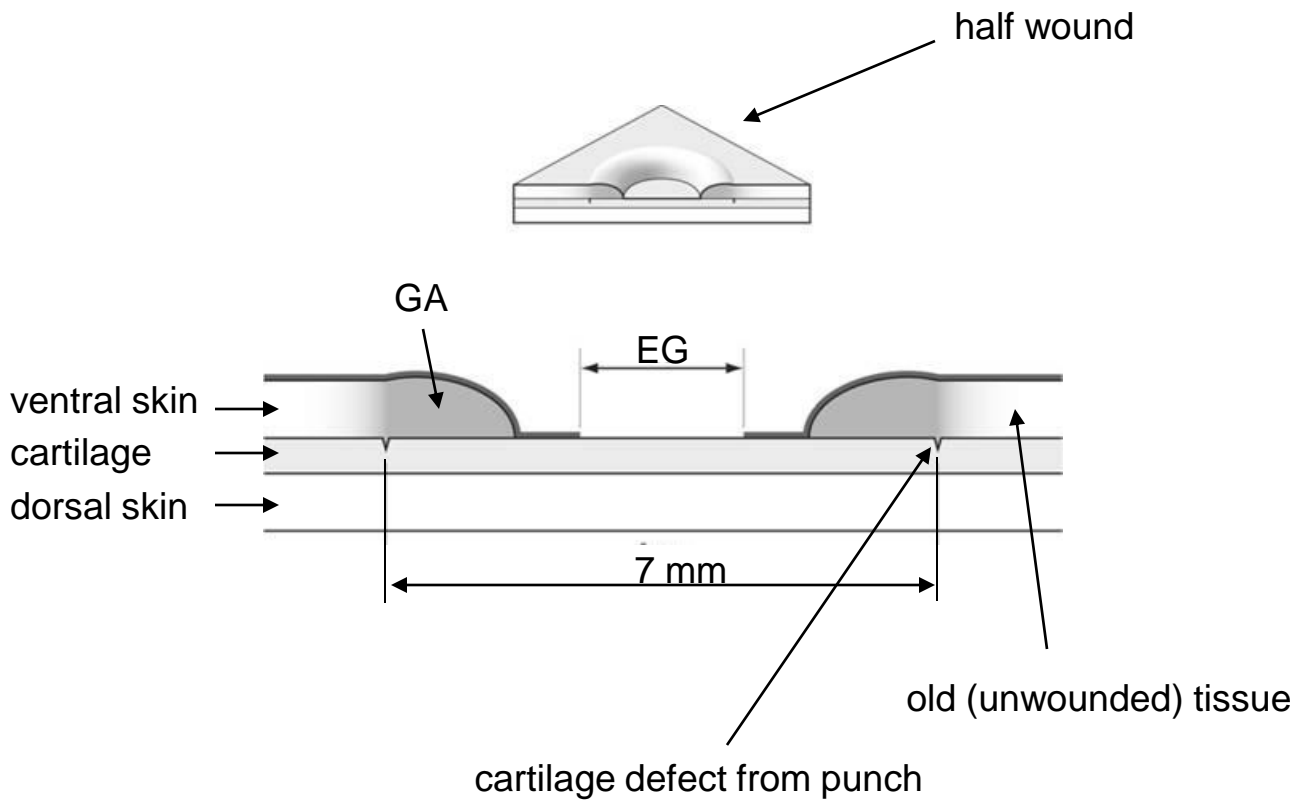

Supplemental Figure S3

Supplement: Figure S3 — Schematic drawing of rabbit wounds and histological analysis. EG, epithelial gap; GA, granulation area. (PDF) [file pone.0055640.s003.pdf]

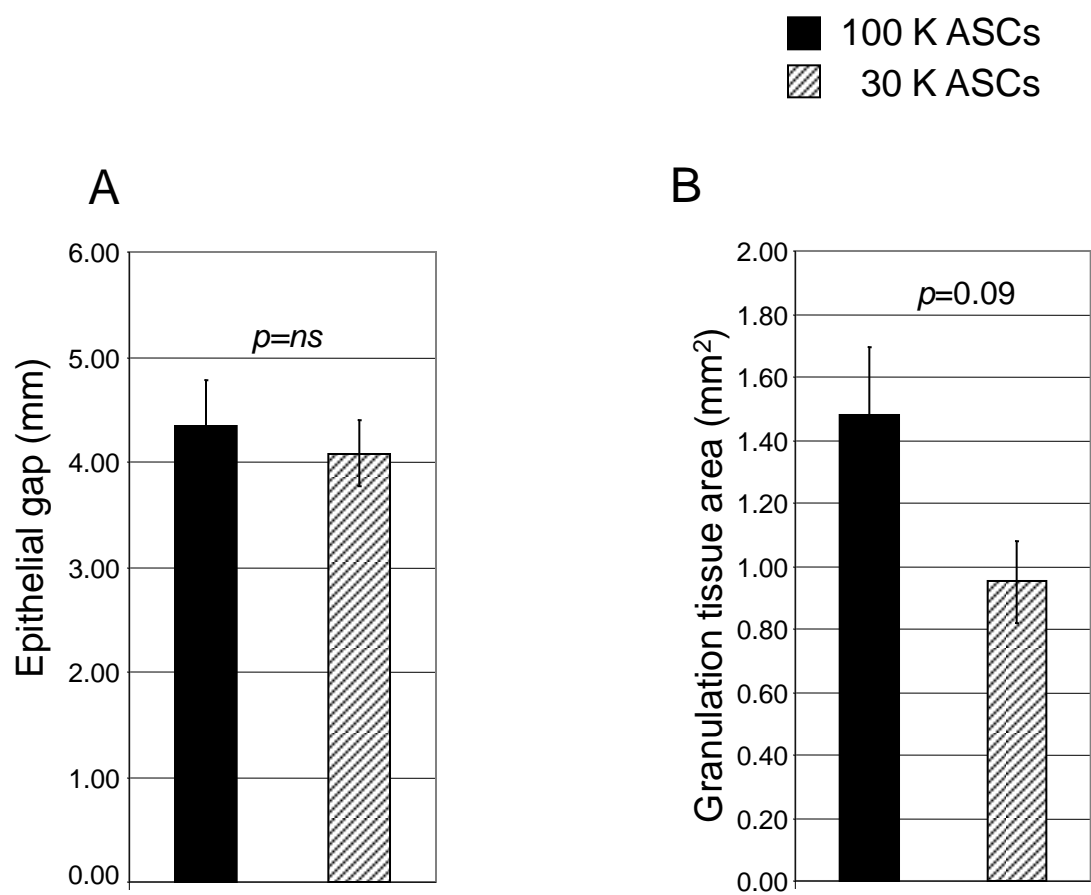

Supplemental Figure S4

Supplement: Figure S4 — Histological quantification of ASCs treated wounds. 1×10^5^ ASCs were delivered to 7 mm wounds on one ear and 3×10^4^ ASCs were delivered to wounds on the contralateral ear of rabbits. Wounds were harvested at POD7 and epithelial gap (A) and granulation tissue area (B) were measured (n = 11 for 1×10^5^ ASCs & n = 12 for 3×10^4^ ASCs). N represents the total number of wounds from two rabbits. Data shown as mean + SEM. ns = not significant. (PDF) [file pone.0055640.s004.pdf]

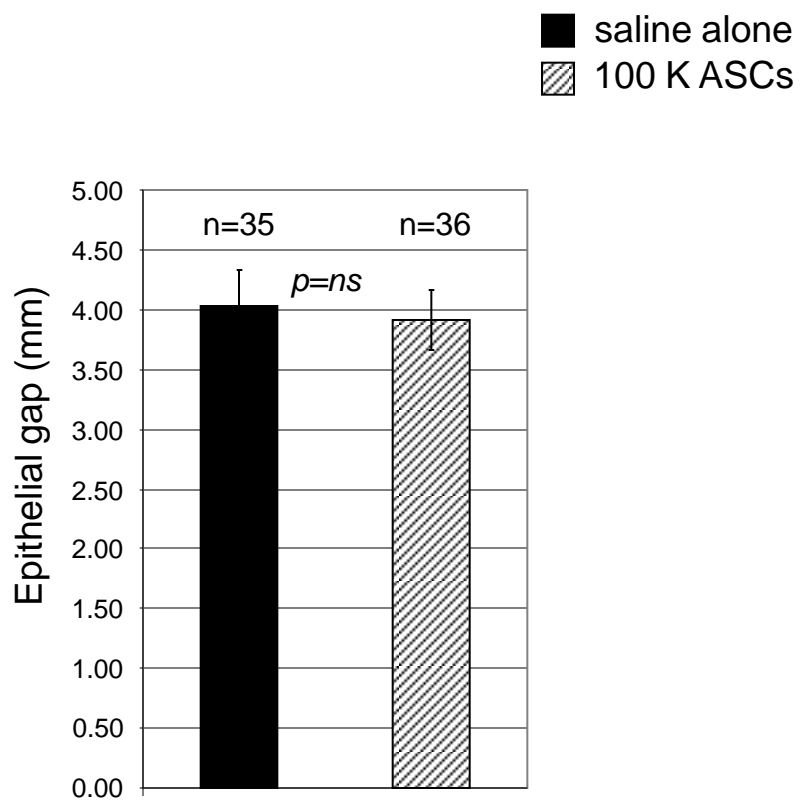

Supplemental Figure S5

Supplement: Figure S5 — Measurement of epithelial gap of ASCs treated wounds. A total of 1×10^5^ ASCs were delivered to 7 mm wounds on one ear. In the contralateral ear, PBS alone was delivered as a control. Wounds were harvested at POD7 and epithelial gap was measured. Data shown as mean + SEM. n = 35 for saline & n = 36 for ASCs. N represents the total number of wounds from six rabbits. ns = not significant. (PDF) [file pone.0055640.s005.pdf]

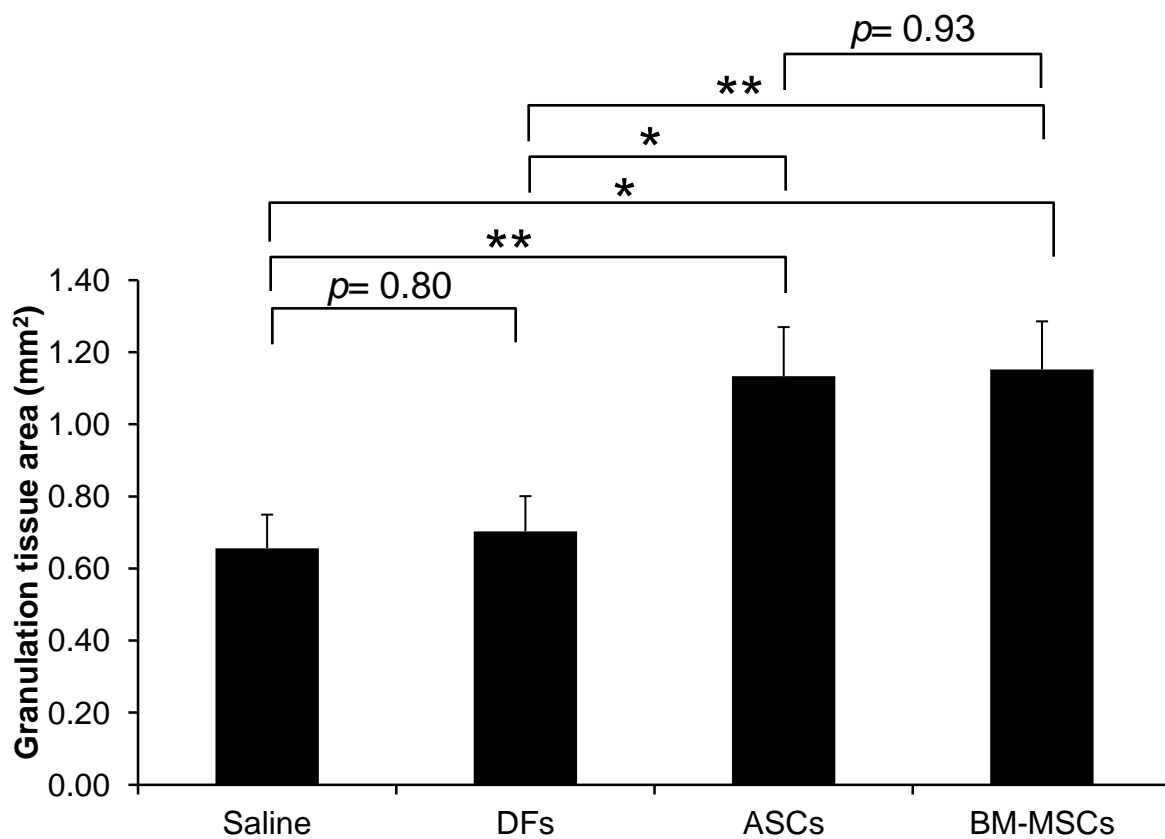

Supplemental Figure S6

Supplement: Figure S6 — Comparison of the effect of ASCs, BM-MSCs, and DFs on wound repair. The granulation tissue measurement data at POD7 wounds in Figure 3 was re-analyzed by the ANOVA with post-hoc analysis. Number of wounds analyzed; n = 69 for saline, n = 20 for BM-MSCs, n = 36 for ASCs, n = 24 for DFs. N represents the total number of wounds from fourteen (saline), six (ASCs), or four (DFs & DM-MSCs) rabbits. Data shown as mean + SEM. *p<0.05, **p<0.01. (PDF) [file pone.0055640.s006.pdf]

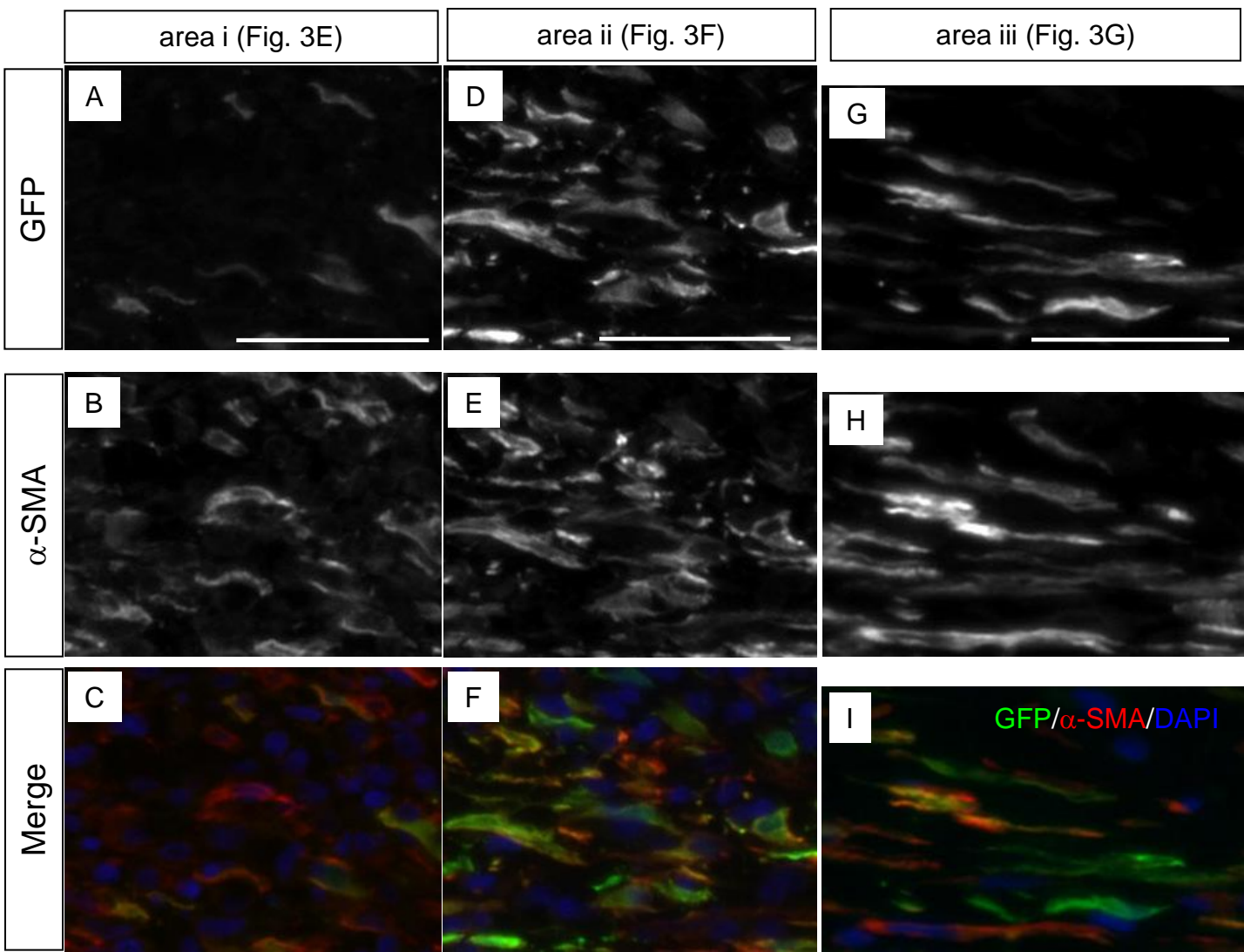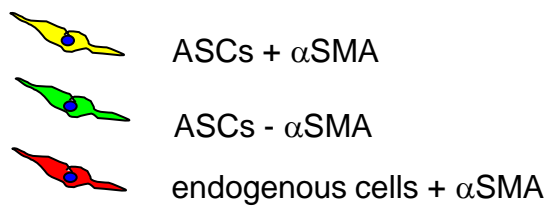

Supplemental Figure S7

Supplement: Figure S7 — Analysis of α-SMA expressing cells in wounds. Chicken anti-GFP and mouse anti-α-SMA antibodies were used to detect GFP and α-SMA. Nuclei were stained with DAPI. GFP (A, D, G), α-SMA (B, E, H), and merged (C, F, I) images in Figure 4E, 4F, 4G were shown. Endogenous cells and transplanted ASCs which express α-SMA showed red and yellow color, respectively, in the merged images (C, F, I). Transplanted ASCs which do not express α-SMA showed green color in the merged images. Scale bars: 50 µm. (PDF) [file pone.0055640.s007.pdf]

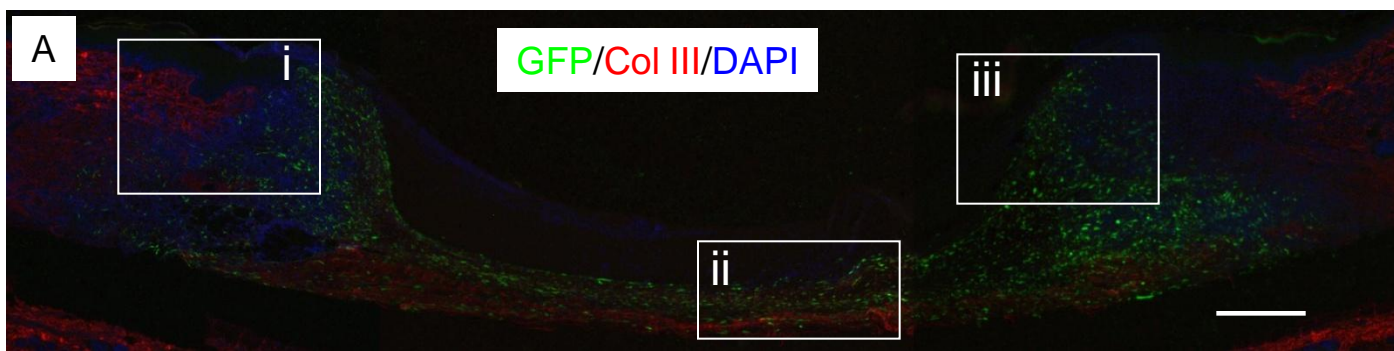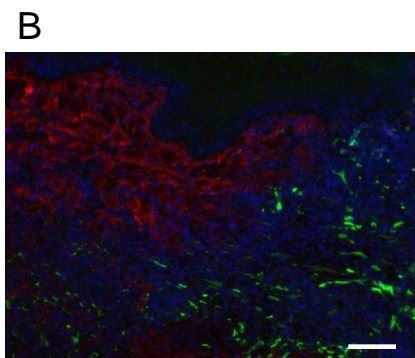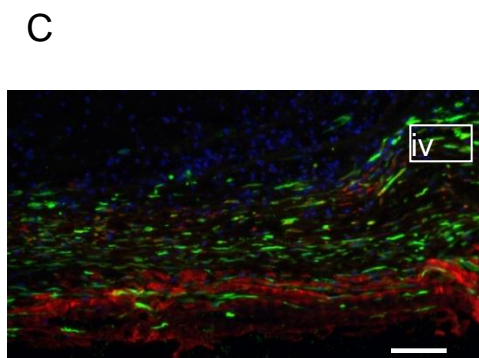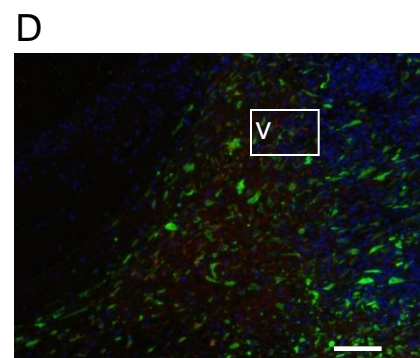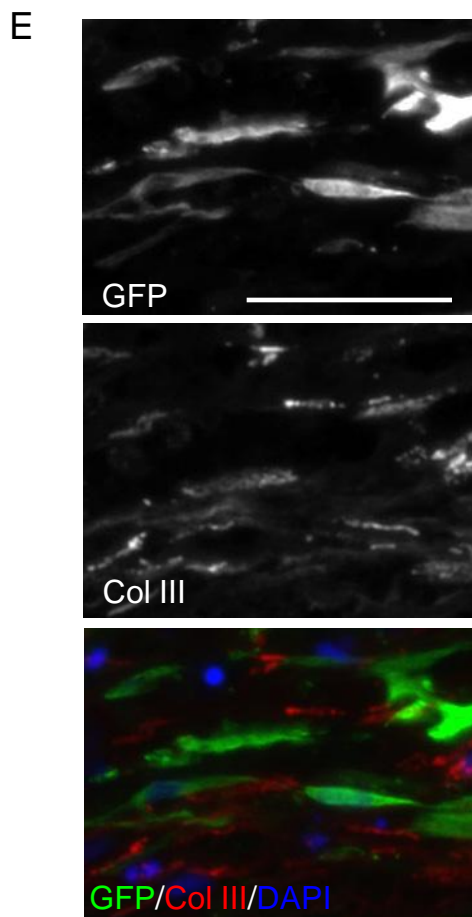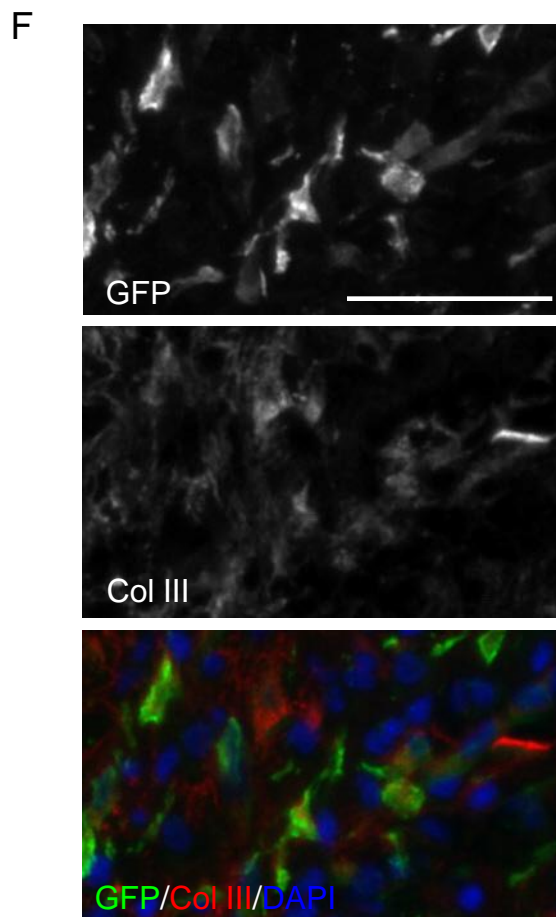

Supplemental Figure S8

Supplement: Figure S8 — Expression of collagen III (Col III) in wounds. GFP-expressing ASCs were analyzed 7 days after transplantation in wounds. Chicken anti-GFP and mouse anti-Col III antibodies were used to detect GFP (green) and Col III (red). Nuclei were stained with DAPI. (A): Low magnification of wounds. (B-D): Higher magnifications of the indicated regions in A (white squares; labeled as i, ii, iii). (E–F): Higher magnifications of the indicated regions in C and D (white squares; labeled as iv and v). Merged images of Col III and GFP were shown. Scale bars: 500 µm (A), 100 µm (B, C, D), 50 µm (E, F). (PDF) [file pone.0055640.s008.pdf]

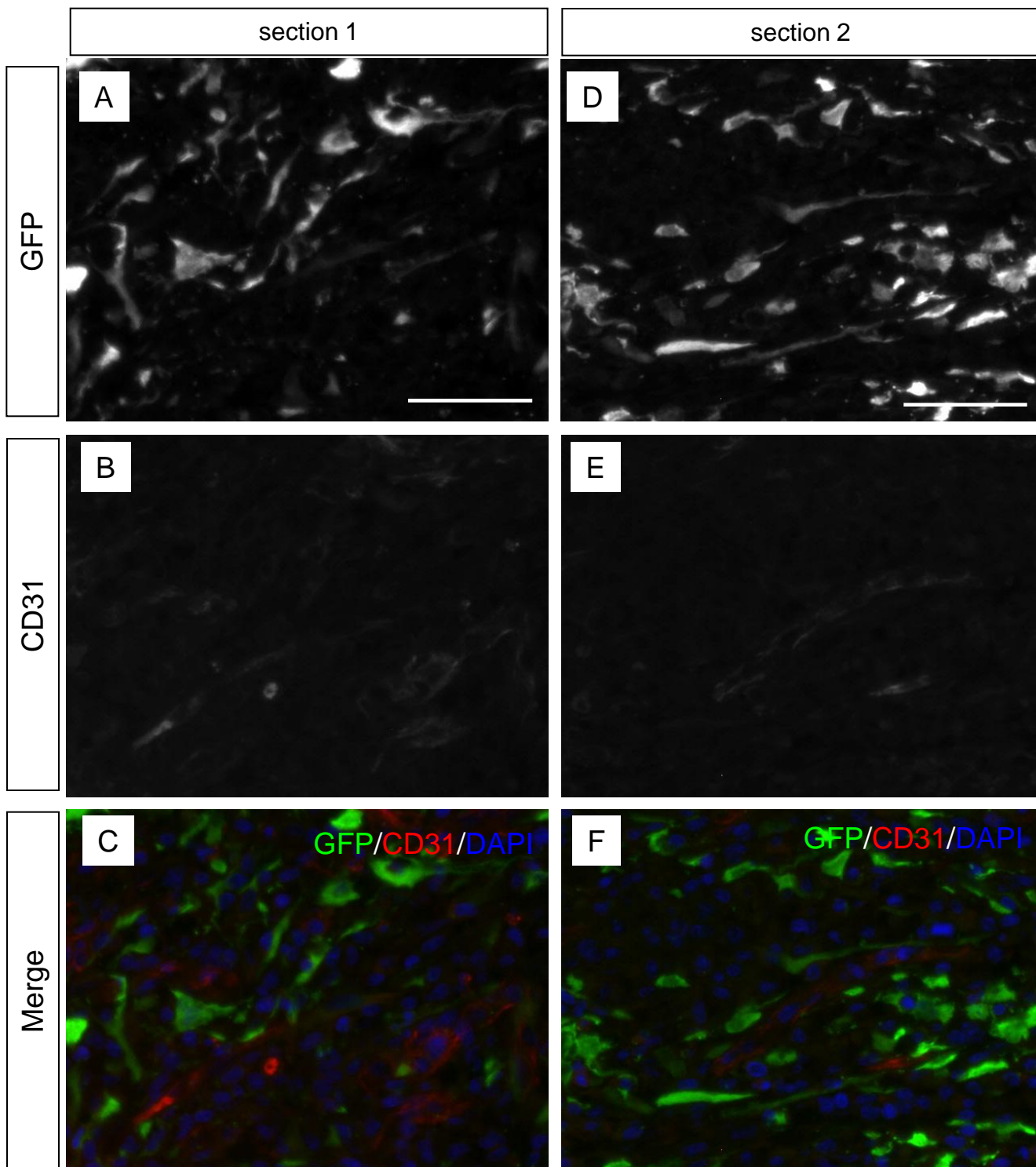

Supplemental Figure S9

Supplement: Figure S9 — Analysis of expression of CD31 (PECAM-1) in transplanted ASCs. GFP-expressing ASCs were analyzed 7 days after transplantation in wounds. Chicken anti-GFP (A, D) and mouse anti-CD31 (B, E) antibodies were used to detect GFP and CD31. Nuclei were stained with DAPI. Co-expression of CD31 and GFP was not detected in the merged images (C, F). Two examples, section 1 and section 2, from the same wound were shown. Scale bar; 50 µm. (PDF) [file pone.0055640.s009.pdf]

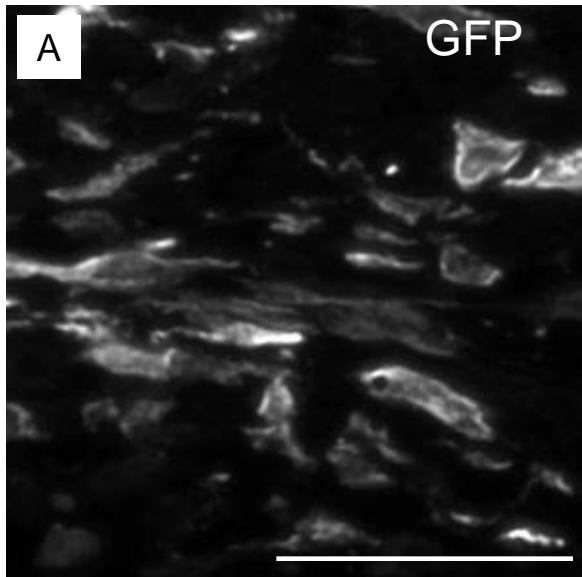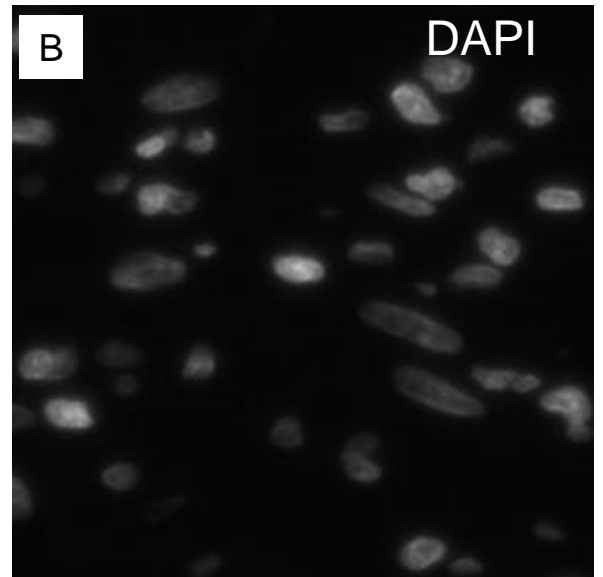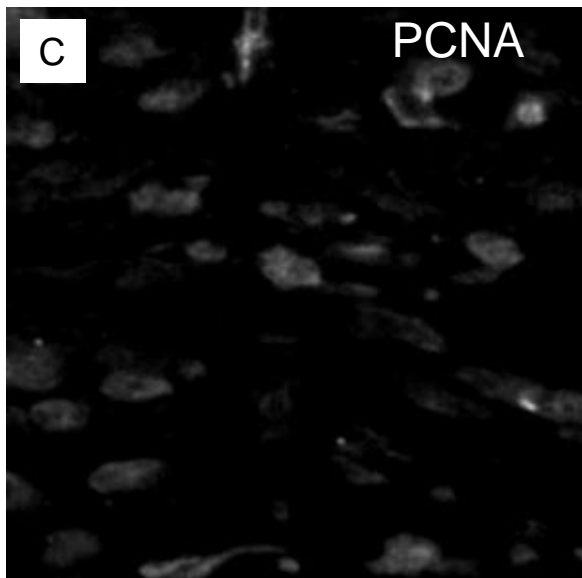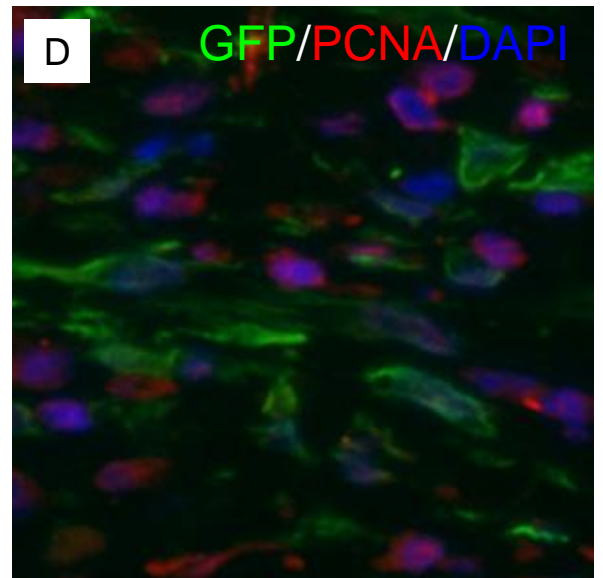

Supplemental Figure S10

Supplement: Figure S10 — Transplanted ASCs proliferate in wounds. GFP-expressing ASCs were analyzed 7 days after transplantation in wounds. Chicken anti-GFP (A) and mouse anti-PCNA (C) antibodies were used. Nuclei were stained with DAPI (B). Merged image was shown in D. Scale bars: 50 µm. (PDF) [file pone.0055640.s010.pdf]
